# Cost-effectiveness of artificial intelligence monitoring for active tuberculosis treatment: A modeling study

**DOI:** 10.1371/journal.pone.0254950

**Published:** 2021-07-21

**Authors:** Jonathan Salcedo, Monica Rosales, Jeniffer S. Kim, Daisy Nuno, Sze-chuan Suen, Alicia H. Chang

**Affiliations:** 1 Department of Pharmaceutical and Health Economics, School of Pharmacy, University of Southern California, Los Angeles, California, United States of America; 2 Leonard D. Schaeffer Center for Health Policy and Economics, University of Southern California, Los Angeles, California, United States of America; 3 Los Angeles County Department of Public Health, Office of Health Assessment and Epidemiology, Los Angeles, California, United States of America; 4 Department of Preventive Medicine, Keck School of Medicine, University of Southern California, Los Angeles, California, United States of America; 5 Los Angeles County Department of Public Health, Tuberculosis Control Program, Los Angeles, California, United States of America; 6 Daniel J. Epstein Department of Industrial and Systems Engineering, University of Southern California, Los Angeles, California, United States of America; URCEco Ile de France Hopital de l’Hotel Dieu, FRANCE

## Abstract

**Background:**

Tuberculosis (TB) incidence in Los Angeles County, California, USA (5.7 per 100,000) is significantly higher than the U.S. national average (2.9 per 100,000). Directly observed therapy (DOT) is the preferred strategy for active TB treatment but requires substantial resources. We partnered with the Los Angeles County Department of Public Health (LACDPH) to evaluate the cost-effectiveness of AiCure, an artificial intelligence (AI) platform that allows for automated treatment monitoring.

**Methods:**

We used a Markov model to compare DOT versus AiCure for active TB treatment in LA County. Each cohort transitioned between health states at rates estimated using data from a pilot study for AiCure (N = 43) and comparable historical controls for DOT (N = 71). We estimated total costs (2017, USD) and quality-adjusted life years (QALYs) over a 16-month horizon to calculate the incremental cost-effectiveness ratio (ICER) and net monetary benefits (NMB) of AiCure. To assess robustness, we conducted deterministic (DSA) and probabilistic sensitivity analyses (PSA).

**Results:**

For the average patient, AiCure was dominant over DOT. DOT treatment cost $4,894 and generated 1.03 QALYs over 16-months. AiCure treatment cost $2,668 for 1.05 QALYs. At willingness-to-pay threshold of $150K/QALY, incremental NMB per-patient under AiCure was $4,973. In univariate DSA, NMB were most sensitive to monthly doses and vocational nurse wage; however, AiCure remained dominant. In PSA, AiCure was dominant in 93.5% of 10,000 simulations (cost-effective in 96.4%).

**Conclusions:**

AiCure for treatment of active TB is cost-effective for patients in LA County, California. Increased use of AI platforms in other jurisdictions could facilitate the CDC’s vision of TB elimination.

## Background

The United States has one of the lowest tuberculosis (TB) case rates in the world, but there is still considerable progress to be made before TB can be eliminated from the US, with over 9,000 cases reported in 2017 [1]. One of the challenges continues to be considerable geographical heterogeneity; for instance, the TB case rate in Los Angeles County (LAC) (5.7 cases per 100,000 population) is significantly higher than the U.S. national average (2.9 cases per 100,000 population). In 2016, there were 550 confirmed TB cases in LAC [2]. LAC had the highest number of TB cases and the 10^th^ highest TB incidence rate among California’s 61 health jurisdictions. We therefore focus on LAC in the present study on technology to improve TB treatment.

Directly observed therapy (DOT) is the preferred strategy for active tuberculosis treatment. DOT is defined as an in person, direct observation by a health care worker of the patient ingesting each dose of medication. DOT directs partial responsibility of treatment to the provider and helps ensure that patients complete an adequate course of TB treatment [3]. While this is an effective method for ensuring TB treatment adherence– 92.6% of TB patients in LAC completed treatment within a year in 2013 –DOT is resource-intensive and requires a high number of nurse-hours per TB patient [4]. Although some DOT sessions are conducted during patients’ clinic visits, in LAC, DOT is mostly carried out in the field, whereby nurses visit TB patients at home to directly observe ingestion of TB drugs which in turn incurs travel expenses and additional time to coordinate home visits [5].

A common alternative to DOT is video directly observed therapy (VDOT), where healthcare professionals observe patients take their medications through a video conferencing software. Many recent studies find success and/or adherence rates with VDOT to be comparable to in-person DOT [6–14]. Some studies have also investigated VDOT costs to the healthcare system in addition to adherence outcomes. Buchman and Cabello (2017) studied traditional DOT vs Skype observed therapy (SOT) in 24 patients from Nassau County, New York [15]. They found cost savings under SOT of $1,008 per-month and $42 per-case per-month. Holzman, Zenilman, and Shah (2018) conducted a prospective pilot study of a VDOT intervention called miDOT (emocha Mobile Health Inc.) in three tuberculosis clinics in Maryland [7]. They followed 28 adult patients receiving active or latent TB treatment. For a standard 6-month treatment, VDOT saved $1,391 per-patient compared to DOT and adherence rates of 98% and 94% for DOT and VDOT, respectively (P = 0.17). Garfein et al. (2018) studied asynchronous VDOT—videos of medication ingestion are recorded by the patient and watched by a health care worker at a later time—versus traditional DOT in five health districts in California (San Diego, San Francisco, Santa Clara, San Joaquin, and Imperial) [8]. They enrolled a total of 174 adults to VDOT and compared outcomes to 159 historical controls treated with DOT. The 6-month treatment cost with VDOT (range $3,031-$3,911) was 32% (range 6%-46%) less than DOT ($3,212-$5,788) across districts.

**Fig 1:**
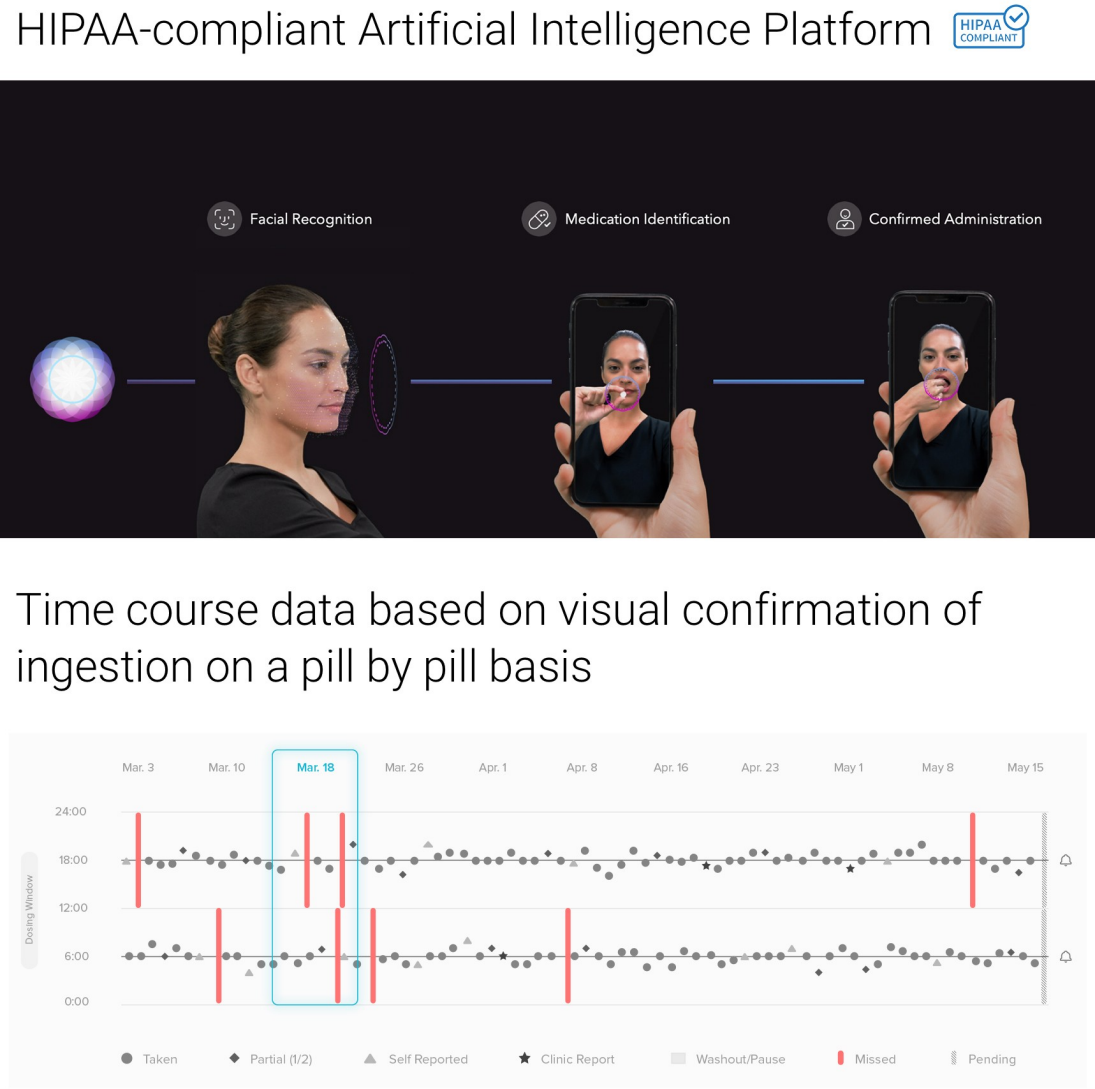
AiCure artificial intelligence platform. Software automatically observes and confirms patient, medication, and ingestion. *HIPAA* Health Insurance Portability and Accountability Act. Source: AiCure, LLC (New York, NY) with written permission to use and adapt.

Recent technological advances have introduced further innovations in this area–artificial intelligence (AI)-based software that uses computer vision and machine learning to confirm the patient, the drugs, and ingestion in real time, further reducing health worker burden. However, there is still limited data on the efficacy of AI-based monitoring methods for TB treatment adherence, and no prior studies to our knowledge have evaluated its cost-effectiveness. In this study, we examine both for a cohort of pulmonary TB patients tracked by the Los Angeles County Department of Public Health (LACDPH) using AiCure (New York, NY), an AI platform that uses computer vision and machine learning to automatically confirm the patient, the drugs, and ingestion in real time. Under AiCure, failure in any one of these criteria leads to an email or SMS text message alert sent to a nurse (see Fig 1 for illustration) who then follows up with the patient in real time. Patients can access AiCure using a smart phone application which uses the device camera to record medication consumption; the platform includes the option to have the video sent to nurses to review later. This platform is currently used in drug development trials and population health settings, across multiple therapeutic areas including neuroscience, cardiovascular, and infectious disease [16–18]. Additional details on AiCure are provided in the online S1 File. By focusing on LAC, a large metropolitan area with high TB morbidity and substantial patient heterogeneity, we hope to provide insight into the cost-effectiveness of AI-based treatment monitoring platforms for a critical TB population in the U.S.

## Methods

### Model overview

We developed a cohort Markov transition state model in Microsoft Excel (Microsoft Corporation, Redmond, WA) to compare cost and treatment completion outcomes of traditional in-person DOT versus AiCure for active TB patients undergoing treatment in LAC. The Markov model tracked monthly TB outcomes for patients over 16 total months, the longest duration on treatment we observed in our data. At each month, patients either continued treatment, defaulted, or successfully completed their treatment; these probabilities were calculated from deidentified LACDPH surveillance data for active TB patients in one of the LACDPH clinics at Pacoima, Los Angeles, CA. Both DOT and AiCure patients were assumed to undergo a standard treatment regimen with an average of 11 observed doses per month. This average reflected the local practice of using both a daily TB treatment regimen as well as a twice a week treatment regimen, according to patient and provider preference. We assumed patients initially unsuccessful on AiCure would be moved to DOT, as is common in clinical practice. We considered patients unsuccessful on DOT as lost to follow-up. Patients must have had a confirmed diagnosis, been without multidrug-resistance (MDR), and been HIV negative to be included in the study population. Our cost-effectiveness study was ruled exempt from Institutional Review Board (IRB) review by the University of Southern California IRB. The pilot research study using AiCure for patients with TB was reviewed and approved by the LACDPH IRB. All experiments were performed in accordance with relevant guidelines and regulations. Informed written consent was obtained from all subjects in the pilot study or, if subjects were under 18, from a parent and/or legal guardian.

Costs by treatment arm, including personnel, technology, and licensing fees, were provided by the LACDPH. We obtained drug costs from the Veterans Affairs Federal Supply Schedule [19]. We estimated total costs on treatment (2017, USD) and total quality-adjusted life years (QALYs) by treatment arm. Our outcomes of interest included incremental cost-effectiveness ratios (ICER) and net monetary benefits (NMB) at various willingness to pay (WTP) values to compare AiCure to DOT. Outcomes were evaluated from the United States societal perspective. To address uncertainty and assess robustness of base results, all parameters were fit to probability distributions and varied in deterministic (DSA) and probabilistic sensitivity analysis (PSA).

### Data

Patients and nurses were trained to use the AiCure software in a pilot study of 43 active TB patients in Pacoima, Los Angeles, CA. In this pilot study, eligible patients were provided with an internet and camera-enabled smart phone and trained to record their doses using the software. This data has not been published elsewhere, and we present it here for the first time. Our final analysis sample for the AiCure arm of our model included 43 patients confirmed during 2015 to 2017 who either began and completed or began and moved/defaulted from active TB treatment. Mean age at diagnosis in this sample was 48.4 years, with 21% of patients having multiple comorbidities, 21% with diabetes, and 76% with pulmonary TB disease (Table 1).

**Table 1:** Summary statistics by treatment arm.

	DOT		AiCure		
	Mean	SD	Mean	SD	*P*-value
**Demographics**					
Age (years)	50.9	(17.07)	48.4	(19.84)	0.530
Year confirmed	2013.5	(0.50)	2016.7	(0.47)	0.001
**Demographics, cont.**	**No.**	**Proportion**	**No.**	**Proportion**	***P*-value**
Male	45	(0.63)	22	(0.65)	0.895
Previous treatment for TB	0	(0.00)	2	(0.06)	0.039
PZA drug resistance	0	(0.00)	1	(0.03)	0.147
*Race/Ethnicity*					
White	36	(0.51)	19	(0.56)	0.619
Asian	33	(0.46)	14	(0.41)	0.609
Black	1	(0.01)	0	(0.00)	0.487
Other	1	(0.01)	1	(0.03)	0.591
Hispanic	32	(0.45)	16	(0.47)	0.848
*Country of birth*					
United States	2	(0.03)	3	(0.09)	0.176
Mexico	23	(0.32)	7	(0.21)	0.210
Philippines	25	(0.35)	7	(0.21)	0.128
Other	21	(0.30)	17	(0.50)	0.042
Years since arrival in USA	15.0	(11.82)	20.2	(13.78)	0.073
**Comorbidities**					
Pulmonary complications	56	(0.79)	26	(0.76)	0.781
HIV positive	0	(0.00)	0	(0.00)	.
Diabetic	20	(0.28)	7	(0.21)	0.406
Renal impairment	2	(0.03)	0	(0.00)	0.323
Immunocompromised	4	(0.06)	0	(0.00)	0.158
Pleural complications	3	(0.04)	3	(0.09)	0.342
Bone joint complications	1	(0.01)	1	(0.03)	0.591
Meningitis	6	(0.08)	1	(0.03)	0.290
Comorbid	25	(0.35)	7	(0.21)	0.128
**Risk factors**					
Homeless	0	(0.00)	0	(0.00)	.
Injecting drug user	0	(0.00)	0	(0.00)	.
Non-injecting drug user	0	(0.00)	1	(0.03)	0.149
Risk for alcohol abuse	0	(0.00)	5	(0.15)	0.001
Smoker	10	(0.14)	0	(0.00)	0.021
No risk factors	38	(0.54)	25	(0.74)	0.050
**Observations**	71		43		114

Category totals may not sum to *N* due to missing values. P-values are calculated using two-sided, two-sample t-tests for proportions or means with unequal variances.

We compared these patients with a cohort of patients with active TB treated with DOT in the same clinic, confirmed between 2013 and 2014 (n = 172), the years immediately preceding the AiCure pilot. To best approximate the population that would have been eligible for AiCure, we included only patients who clinicians determined to be eligible for AiCure treatment. Our control final sample included 71 patients previously treated for active TB under DOT. Average age at diagnosis in this sample was 50.9, with 35% having multiple comorbidities, including 28% diabetic and 79% with pulmonary disease (Table 1). We calculated *P*-values using two-sided, two-sample t-tests for proportions or means with unequal variances. No demographic factors including age, gender, or race were statistically significantly different across treatment arms. Although not selected for, the demographic characteristics and proportion of patients with comorbidities in each treatment arm were similar. In both arms most patients had no behavioral risk factors and no major morbidities other than pulmonary complications.

### Costs

For both treatment arms, we calculated drug acquisition costs to be $174.16 per-patient per-month, assuming treatment with the standard regimen of isoniazid (INH), rifampin (RIF), pyrazinamide (PZF), and ethambutol (EMB) [3, 19]. AiCure-specific fees include licensing for $750 per clinic per month up to 50 patients, $1,500 per clinic per month for technical support, a fixed cost of $52 per patient for a phone, and $47.50 per patient per month for phone service. We estimated the remaining costs in the model using Licensed Vocational Nurse (LVN) or Registered Nurse (RN) hourly compensation rates provided by LACDPH (Table 2). Additional details on costs are available in the online S1 File.

**Table 2:** Baseline model parameters.

Description	DOT	AiCure	Source
**General Parameters**			
Patients	100	100	Assumed
Clinic capacity	100	100	Assumed
Doses, monthly	11	11	LACDPH
FDOT proportion	0.69	-	LACDPH
FDOT appointment time, hours	0.83	-	LACDPH
FDOT miles, per appointment	7.60	-	LACDPH
FDOT missed appointment time, hours	0.42	-	LACDPH
FDOT missed appointment frequency, PMPM	2.50	-	LACDPH
CDOT appointment time	0.25	-	LACDPH
CDOT missed appointment time	0.17	-	LACDPH
CDOT missed appointment frequency, monthly	2.60	-	LACDPH
AI dose time	-	0	LACDPH
AI missed dose time	-	0.208	LACDPH
AI missed dose frequency, monthly	-	1.3	LACDPH
AI appointment frequency, monthly	-	1.5	LACDPH
**Baseline Completion Probabilities**^**a**^			
P(Complete | t ≤ 120 days)	0.000	0.000	LACDPH
P(Complete | 120 < t ≤ 150 days)	0.000	0.000	LACDPH
P(Complete | 150 < t ≤ 180 days)	0.000	0.051	LACDPH
P(Complete | 180 < t ≤ 210 days)	0.217	0.306	LACDPH
P(Complete | 210 < t ≤ 240 days)	0.149	0.120	LACDPH
P(Complete | 240 < t ≤ 270 days)	0.050	0.045	LACDPH
P(Complete | 270 < t ≤ 300 days)	0.684	0.524	LACDPH
P(Complete | 300 < t ≤ 330 days)	0.083	0.600	LACDPH
P(Complete | 330 < t ≤ 360 days)	0.091	0.250	LACDPH
P(Complete | 360 < t ≤ 390 days)	0.600	0.333	LACDPH
P(Complete | 390 < t ≤ 420 days)	0.500	0.500	LACDPH
P(Complete | 420 < t ≤ 450 days)	0.500	1.000	LACDPH
P(Complete | 450 < t ≤ 480 days)	1.000	1.000	LACDPH
**Utilities (QALY weights)**			
Active tuberculosis	0.663	0.663	(20, 21)
Healthy, post treatment	0.942	0.942	(20, 21)
**Costs (2017 USD)**			
Licensed vocational nurse, hourly	$35.45	$35.45	LACDPH
Registered nurse, hourly	$79.26	$79.26	LACDPH
Mileage, per mile	$0.54	$0.54	IRS
Licensing per 50 patients, monthly	-	$750.00	LACDPH
Phone purchase	-	$52.00	LACDPH
Phone service, monthly	-	$47.50	LACDPH
Technical support, monthly	-	$1,500.00	LACDPH
Pharmaceutical acquisition, monthly	$174.16	$174.16	(19)

^a^ See S1 Table in S1 File for complete table of continuation, completion, and default probabilities. *LACDPH*, Los Angeles County Department of Public Health; *FDOT*, field directly observed therapy; *CDOT*, clinic directly observed therapy; *PMPM*, per-member per-month; *AI*, AiCure; *QALY*, quality-adjusted life year.

### Health states and treatment progression

**Fig 2:**
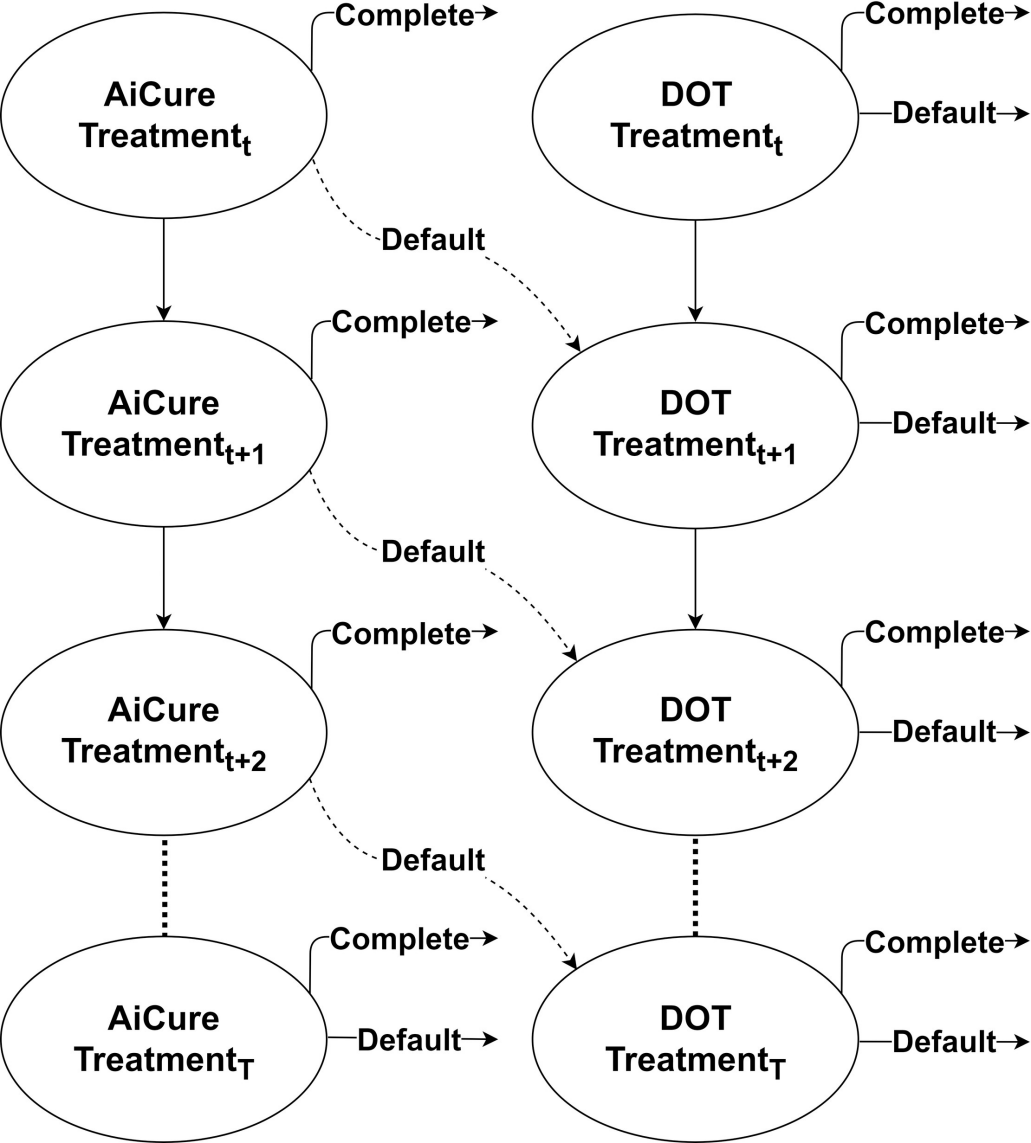
Markov state transition model for AiCure and DOT arms. AiCure patients (left) could default to DOT treatment (right). *DOT* directly observed therapy.

We modeled patient progression from treatment initiation to completion using a Markov model. In the model, individuals were on-treatment, had completed treatment, or had defaulted (Fig 2). Consistent with LACDPH active TB treatment guidelines, we assumed a minimum successful treatment length of five months for all patients [3]. At each 30-day increment from 4–16 months of treatment, patients had some probability of completing or defaulting from treatment on each treatment arm (values found using the LACDPH dataset). Consistent with clinical practice, non-adherent patients on AiCure would be moved to DOT for the remainder of treatment. See S1 Table in S1 File for detailed transition probabilities by arm.

### Health utilities

We identified health-related quality of life (HR-QoL) utilities for each health state from the medical literature. The utility for living with active drug-susceptible TB and taking TB treatment was 0.663 and healthy with past TB treatment was 0.942 [20, 21]. A summary of utility values and sensitivity analysis ranges are provided in Table 2 and S2 Table in S1 File, respectively. We assumed that the health utility for being on AiCure was the same as for DOT, as the only difference is the method of treatment monitoring. We counted defaulting patients as living with active TB for the remainder of the observation period.

### Sensivity analyses

To check for robustness of our results, we conducted deterministic and probabilistic sensitivity analyses. In the scenario in which the base case resulted in a dominant treatment option, the outcome of interest was changed from ICERs to incremental net monetary benefits (INMB) of AiCure with willingness to pay (WTP) of $150,000 per QALY [22]. For one-way deterministic sensitivity analysis, we varied model inputs within their 95% confidence intervals, predicted ranges, or +/- 50% when neither were available. Probabilistic sensitivity analysis involved fitting likely distributions to input parameters and simulating outcomes in 10,000 Monte Carlo repetitions [23–25].

### Scenario analyses

As the number of observed patients on AiCure was relatively small, there exists uncertainty around the monthly treatment completion rate. We therefore examined several scenario analyses to identify thresholds for which AiCure would still be cost-effective even if monthly treatment rates are lower. In the first scenario, we examined what would happen if monthly AiCure completion rates are lower by 5 percentage points per month. We repeated this analysis where AiCure completion rates are lower than observed in our data by 10 percentage points and 15 percentage points.

As a secondary scenario analysis, we also examined the “best case” and “worst case” scenario where the non-transition probability parameters from one-way DSA are at their 95% confidence interval bounds to either make AiCure/DOT perform well or poorly. Outcomes of interest in all scenario analyses were the variation in ICER and cost-effectiveness outcomes for AiCure.

We additionally include a third scenario analysis, a comparison of AiCure to VDOT, where we examine the cost-effectiveness outcomes if additional resources were needed in adopting AiCure in a new location. Any jurisdiction seeking to adopt an AI treatment monitoring system may have nurses monitor patient video records (similar to VDOT) to ensure the AI system is operating correctly. We therefore examine the scenario where nurses must review patient video on the AiCure platform, spending 7 minutes per video.

## Results

Among AiCure patients, average total days on treatment was 252.5 (SD = 69.4, range 94 to 401), slightly higher but not statistically significantly different from the DOT average of 247.1 (SD = 89.1, range 8 to 456) (p = 0.729) (Table 3). Among those who successfully completed treatment, mean days were 258.9 (SD = 64.4) and 270.5 (SD = 67.4) for AiCure and DOT, respectively (p = 0.395).

**Table 3:** Result statistics by treatment arm.

	DOT		AiCure		
	No.	Prop.	No.	Prop.	*P*-value
**Treatment status counts**					
Completed	62	(0.87)	38	(0.88)	0.869
Moved	6	(0.08)	3	(0.07)	0.777
Defaulted^a^	3	(0.04)	2	(0.05)	0.914
**Days on treatment for TB**	**Mean**	**SD**	**Mean**	**SD**	***P*-value**
Total	247.1	(89.07)	252.5	(69.36)	0.729
Completers	270.5	(67.39)	258.9	(64.44)	0.395
Defaulters^a^	124.0	(3.00)	130.5	(51.62)	0.888
**Observations**	71		43		114

^a^ Default is defined as patients who refused continued treatment, were lost to follow-up, or went off protocol for medical reasons during our observation period. Category totals may not sum to *N* due to missing values. Movers were excluded from transition probability calculation. P-values are calculated using two-sided, two-sample t-tests for proportions or means with unequal variances.

Using the model, we found that AiCure results in greater benefits at a lower cost over DOT. In our base case analysis, AiCure cost an average of $2,668 per-patient, while DOT cost 83% more, at $4,894 per-patient over the 16-month horizon (Table 4). Our model results indicated AiCure produced 1.05 QALYs per patient over the 16-month time horizon and DOT produced 1.03 QALYs.

**Table 4:** Model results under base case and scenario analyses.

Scenario	AiCure		DOT		AiCure vs. DOT		
	Cost (2017 USD)	QALYs	Cost (2017 USD)	QALYs	Cost (2017 USD)	QALYs	ICER (2017 USD)
Base case	$2,668	1.05	$4,894	1.03	-$2,226	0.02	AI Dominant
AI 5% worse^a^	$2,860	1.03	$4,894	1.03	-$2,034	0.00	AI Dominant
AI 10% worse^a^	$3,011	1.02	$4,894	1.03	-$1,882	-0.01	$244,651 (DOT)
AI 15% worse^a^	$3,164	1.01	$4,894	1.03	-$1,730	-0.02	$89,889 (DOT)
AI WC/DOT BC	$5,278	1.08	$1,607	1.08	$3,672	0.01	$433,646 (AI)
AI BC/DOT WC	$2,883	1.01	$10,485	0.98	-$7,603	0.03	AI Dominant

^a^ x% worse implies an x percentage-point decrease in all monthly completion probabilities up to 15 months, with a floor of zero. The worst/best scenario involves model one-way sensitivity parameters tested at their 95% confidence interval lower or upper bounds. All costs and QALYs are reported per-patient. *AI*, AiCure; *DOT*, directly observed therapy; *ICER*, incremental cost-effectiveness ratio, *QALY*, quality-adjusted life year; *BC*, best case; *WC*, worst case.

Incrementally, AiCure patients gain an additional 0.02 QALYs at cost savings of $2,226 over DOT. At WTP of $50K, $100K, and $150K per QALY, resulting in incremental NMB per patient under AiCure versus DOT that were all above zero ($3,142, $4,057, and $4,973 respectively).

In univariate DSA, no variation in the model inputs within reasonable ranges resulted in a negative incremental NMB for AiCure. The model was most sensitive to the number of doses taken per-member per-month (11 in the base case). Varying this parameter between 5.5 and 16.5 doses per-month resulted in an NMB range of $3,633 to $6,313 WTP of $150K/QALY. The model was also highly sensitive to hourly wage for licensed vocational nurses (LVN) of $35.45; varying this value (+/- 50%) resulted in an NMB range of $3,885 to $6,061 (see tornado diagram in S1 Fig in S1 File). These results suggest much of the cost savings from AiCure can be attributed to the significant decrease in personnel costs required per dose. As DOT treatment requires LVNs or other skilled personnel to engage in significant travel, the trip frequency/distance and hourly rate of these individuals drives large cost differences.

**Fig 3:**
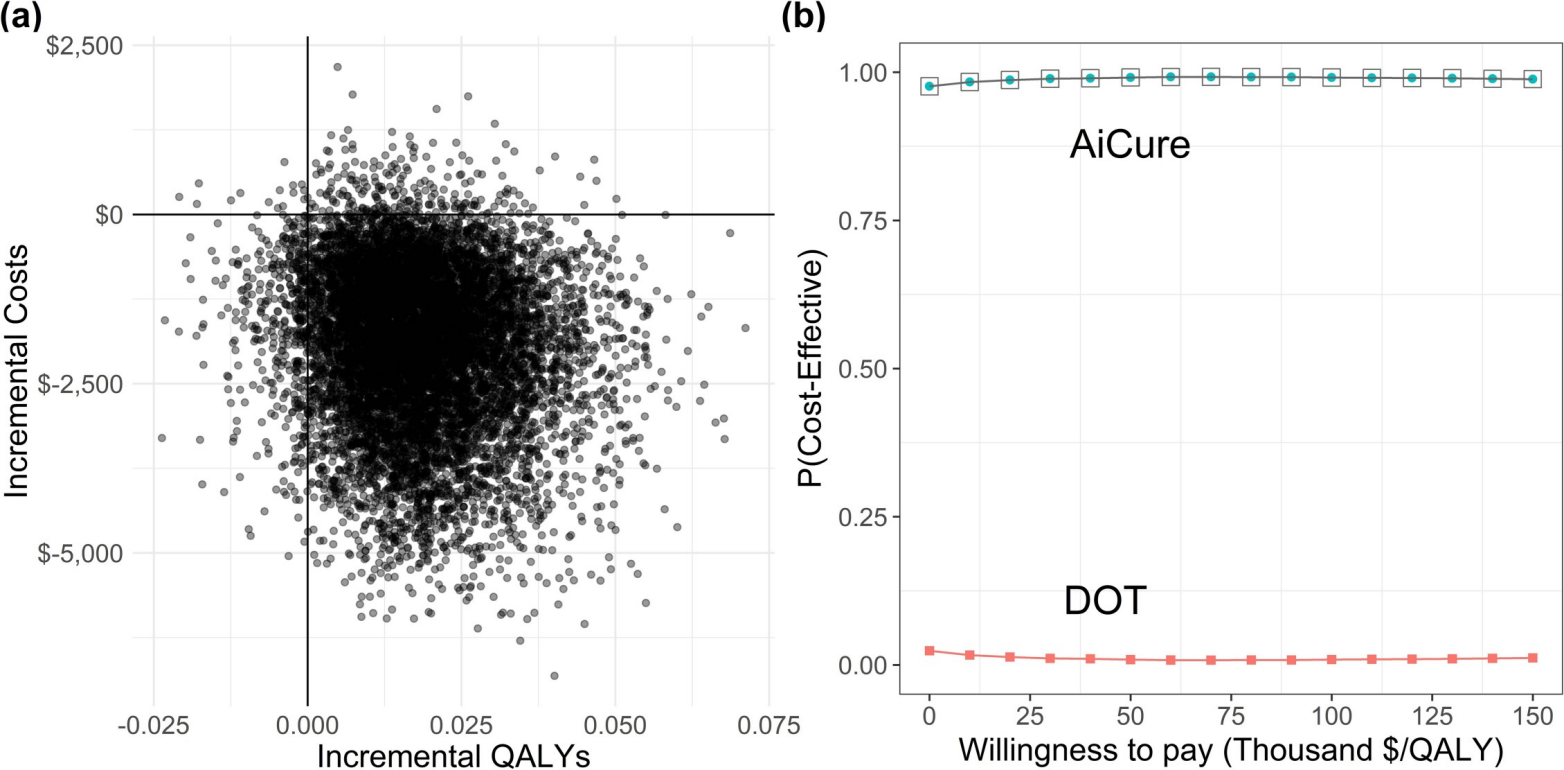
Probabilistic sensitivity analysis results. Panel a: Incremental values per-patient of AiCure relative to DOT. Panel b: Cost-effectiveness acceptability curve (CEAC) by treatment arm. *DOT* directly observed therapy, *QALY* quality-adjusted life year.

In PSA, AiCure produced more QALYs per patient at a lower cost than DOT in 93.5% of 10,000 simulations (Fig 3). At WTP/QALY of $50K, $100K, and $150K, the probability of AiCure being cost-effective is 95.3%, 95.9%, and 96.4%, respectively. These results suggest the base scenario is robust to univariate and probabilistic variations in the input parameters. However, we find there is some probability that DOT is the more cost-effective choice as the WTP for a QALY decreases (see cost-effectiveness acceptability curve [CEAC] in Fig 3B).

Our scenario analyses also suggest that the most a clinic could lose by adopting AiCure would be relatively small (Table 4). In the “worst case” scenario, we find that AiCure would not be cost-effective, generating 0.01 more QALYs per patient for $3,672 more dollars than DOT (AiCure ICER of $433,646). On the other hand, clinics could stand to gain much more by adopting AiCure: in the “best case” scenario, we find that AiCure could generate 0.03 more QALYs per patient for $7,603 less costs than DOT. The difference in health outcomes are quite small in both the “best” and “worst” cases, indicating that patient health would not be substantially affected with or without AiCure, although the financial impact could be large. See the online S1 File for additional details on our primary and secondary scenario analyses.

In the third scenario analysis, we compared AiCure to VDOT, where nurses monitor patient video records to ensure the AI system is operating correctly. We find that even with this additional monitoring the system would still be cost saving. If 7 minutes were spent reviewing each dose (as observed in LA County’s system after AiCure’s initial adoption during implementation of the parent research study), AiCure still saved $1,517 per patient relative to DOT. This 7-minute figure per-dose is included in the “worst case” scenario analysis previously described and is comparable to figures found for VDOT in previous studies. AI monitoring systems could potentially save a few hundred dollars per patient compared to VDOT systems. Consistent with prior studies, we estimate that there would be a cost savings of roughly $1,500 per patient if nurses reviewed video of each dose, which is about $700 less savings than using the AI monitoring system in our base case [7, 8, 15]. This could amount to substantial savings for large health systems if AI monitoring was used at scale.

## Discussion

The data from LAC patients suggest that AiCure would be an effective method of reducing nurse and patient burden and achieving similar levels of DOT success. Patients on AiCure completed treatment in a statistically indistinguishable amount of time as DOT (p = 0.729) while costing $2,226 less, on average. Sensitivity analysis results showed that despite the uncertainty around completion times due to the limited number of patients in the LAC data, AiCure would be the cost-effective strategy over 95% of the time with willingness-to-pay levels over $50,000 per QALY. Even in cases where DOT was cost-effective over AiCure, the largest amount DOT could save over AiCure over all possible input values was modest ($3,672 saved per-patient) relative to the largest savings for AiCure over all possible input values ($7,603 saved per-patient).

However, these results may not be generalizable to all TB clinics and all patients. The cohorts used for comparison in this study were not perfectly statistically similar across all characteristics, constitute a small sample of all patients, and indeed may not be fully representative of the LAC TB patient population. We limited our analyses to non-drug resistant pulmonary TB cases only, as most TB cases in LAC are not complicated by extra-pulmonary TB or drug resistance–in 2015, 71.5% of TB patients had exclusively pulmonary disease. These would also be the most likely patients to be first shifted to an AI Platform, as their treatment regimens are relatively simpler. The cost savings and treatment outcomes in this analysis may not apply to a patient population with more complicated case histories or comorbidities, nor to health programs less geographically sprawling than those in LAC–the costs included those of LVN travel (an average of $35.45 per hour and 54 cents per mile in the base analysis). However, while cost-savings may be lower in smaller counties, variation in travel costs or nurse wages in the sensitivity analyses did not lead to DOT becoming cost-effective over AiCure in any scenario.

We also wish to acknowledge the assumptions in our model. We do not consider in the model possible side effects or delays in appropriate care due to a reduction in frequency of patient-nurse contacts, as we assume that patients’ monthly clinic visits are sufficient to address any treatment complications. We note that our base case may underestimate the true cost savings of AI treatment monitoring platforms for patients with more than 2–3 DOT field or clinic appointments per week; an increased frequency of clinic or field visits would only widen cost differences through necessary LVN and RN personnel hours. Recent ATS/CDC TB treatment guidelines recommend the use of daily rather than intermittent (such as twice or thrice weekly) TB therapy [26, 27]. This recommendation has been difficult to implement fully due to staff capacity. As LACDPH increasingly meets this standard, we would expect the true cost savings of AiCure to increase. We do not consider transmission in this analysis, with the assumption that both intervention arms effectively limit spread of TB soon after the patient starts treatment. We also assume that all patients who complete care would have roughly equivalent quality of life post-treatment whether on DOT or AiCure, and we therefore only consider the 16-month treatment for the time horizon.

Despite these limitations, our analysis suggests that AiCure would be a cost-saving alternative to DOT with no sacrifice in quality of care, provided it is offered to uncomplicated non-drug resistant pulmonary TB cases. Increased access to and implementation of AI treatment monitoring systems could potentially reduce the costs of already resource-constrained public health systems and free nurses to perform skilled tasks instead of traveling to patients or reviewing videos of patient to ensure adherence. These results suggest that the AiCure platform may be a reasonable option to consider for patients beyond those in the pilot study.

## Conclusions

The evidence from this pilot study suggests that AI treatment monitoring platforms for TB may be worth investing in as cost-savings under typical operation should be substantial compared to DOT without degradation of patient outcomes. Our findings contribute to a growing literature on the value and application of artificial intelligence in medicine to improve clinical decision-making, implement efficiencies, and drive behavioral modification across large patient populations, especially with regards to remote treatment options for conditions which disproportionately affect disadvantaged populations in the US. Future work may look at the heterogeneity in outcomes by patient population and the value of conducting additional, larger-scale multi-arm studies to reduce uncertainty around efficacy and cost estimates.

## Supporting information

S1 File Supplementary information for cost-effectiveness of artificial intelligence monitoring for active tuberculosis treatment: A modeling study. (DOCX)

## Abbreviations

AI: artificial intelligence
ATS: American Thoracic Society
BC: best case
CA: California
CDC: Centers for Disease Control and Prevention
CEAC: cost-effectiveness acceptability curve
DOT: directly observed therapy
DSA: deterministic sensitivity analyses
EMB: ethambutol
HIV: human immunodeficiency virus
HR-QoL: health-related quality of life
ICER: incremental cost-effectiveness ratio
INH: isoniazid
INMB: incremental net monetary benefits
IRB: institutional review board
LA: Los Angeles
LAC: Los Angeles County
LACDPH: Los Angeles County Department of Public Health
LVN: licensed vocational nurse
MDR: multidrug-resistance
NMB: net monetary benefits
NY: New York
PSA: probabilistic sensitivity analyses
PZF: pyrazinamide
QALY: quality-adjusted life year
RIF: rifampin
RN: registered nurse
SD: standard deviation
SI: supplementary information
SMS: short message service
SOT: Skype observed therapy
TB: tuberculosis
US: United States
USA: United States of America
USD: United States dollar
VDOT: video directly observed therapy
WA: Washington
WC: worst case
WTP: willingness to pay
