# Supplementary material for: Cost-effectiveness of artificial intelligence monitoring for active tuberculosis treatment: A modeling study
Source: PLoS One. 2021 Jul 21;16(7):e0254950. doi: 10.1371/journal.pone.0254950 (PMC8294556; doi:10.1371/journal.pone.0254950)
Supplement: S1 File — (DOCX) [file pone.0254950.s001.docx]

**Supplementary Information for Cost-Effectiveness of Artificial Intelligence Monitoring for Active Tuberculosis Treatment: A Modeling Study**

Jonathan Salcedo, Monica Rosales, Jeniffer S. Kim, Daisy Nuno, Sze-chuan Suen and Alicia H. Chang

**AI Treatment Monitoring Platform: AiCure**

AiCure (New York, NY) uses computer vision and machine learning to identify the patient, drug, and ingestion of the drug. All dosing events are date and time stamped on a pill by pill basis. The HIPAA-compliant platform encrypts data that are sent to cloud-based dashboards for real-time computer monitoring and intervention if needed. Alert flags are provided for suspicious activity, non-adherence, and incorrect usage to help notify health workers who can then follow up with the patient. The platform automatically detects medication ingestion and flag complications for nurse review, allowing nurses to focus on cases that need more attention. It is an exception-based system – only doses that are not completed correctly are flagged. The AI platform is available on Android and iOS.

**Personnel costs**

The model assumes all personnel duties are done by either a Licensed Vocational Nurse (LVN) or a Registered Nurse (RN)

Hourly salaries (2017 USD) are provided by LACDPH:

- LVN: $35.45
- RN: $79.26

While other jurisdictions may employ community health workers (CWs) rather than LVNs for field travel, we estimated hourly rates for CWs in LAC over the same period ($34.90) to be very close to our model’s base rate for LVNs ($35.45) and fell within the range covered in the sensitivity analyses.

**Drug costs**

We calculated drug costs for a standard six-month treatment regimen including a two-month intensive phase, for an average 83kg bodyweight individual. This included isoniazid (INH), rifampin (RIF), pyrazinamide (PZF), and ethambutol (EMB) at prices extracted from the US Veterans Affairs Federal Supply Schedule (VA FSS).

The patient would take PZF+INH+RIF at 22.5mg/kg daily and EMB at 15 mg/kg daily for the first two months at a monthly cost of $435.75. For the remainder of treatment, patients would take INH (5mg/kg) and RIF (10mg/kg) daily for four months ($43.50/mo).

Six-month drug costs for a standard regimen totaled $1045.48. However, since our data shows patients on treatment for an average of over eight months, we calculated a monthly cost of pharmaceutical acquisition by dividing the six-month regimen cost by six ($174.16).

We note that our AiCure pilot only included patients in the continuity phase of treatment, using only INH and RIF at that point. For this reason, patients had already completed the 2 months of INH, RIF, PZA, and EMB beforehand. However, since we are interested in modeling a scenario in which AiCure is used for the entire treatment regimen, we consider drug costs as identical to those for patients on DOT.

**DOT**

Field Appointment

- 69.4% of doses are field, done by LVN
- 7.60 miles driven (one way) per trip, travel cost may be inferred using IRS standard mileage rate of 53.5 cents per mile
- Average appointment takes 35-65 minutes, including travel time
- A missed/rescheduled appointment results in additional 20-30 minutes (happens 3 times per patient per month on average)

In-Person Appointment

- 30.6% of doses are clinic, done by RN
- Average appointment takes 15 minutes
- A missed/rescheduled appointment results in additional 10 minutes (happens 2.6 times per patient per month on average)

**AiCure**

- Review done by RN
- Initial meeting time of 30-40 minutes
- Average time spent per dose 7 minutes
- Additional 10-15 minutes if problems arise (missed dose happens 1.3 times per patient per month on average)
- A missed/rescheduled appointment results in additional 10 minutes
- Patient will have 1-2 clinic appointments per month

**Transition probabilities**

We calculated transition probabilities by using empirical days on treatment by treatment arm. From the data we created thirty-day intervals from four to sixteen months during which patients could: continue, complete, or default from treatment. We calculated these probabilities assuming that any defaulters with missing number of days (excluding patients who moved residence) had the mean default time observed in his or her treatment arm. We used the model to calculate the number of individuals still on treatment at each month starting with the cohort of 100 patients in each treatment arm, from which we could find total costs and health utilities for our cost-effectiveness analysis. This allowed for greater generalizability than calculating the total costs and outcomes for the observed patients in each treatment arm. We fit these transition probabilities for each thirty-day interval to Dirichlet distributions and varied them in probabilistic sensitivity analysis.

**Scenario analyses**

***Primary***

We lowered monthly AiCure completion rates by 5, 10, and 15 percentage points (with a floor of zero) to examine when it would perform worse than DOT. We found that AiCure could have roughly a 5-percentage point decrease in each monthly treatment completion rate from months 6-15 and still achieve the same level of QALYs as the DOT patients. If AiCure patients completed treatment every month at a 5 percentage points lower rate than observed, AiCure would still produce 0.004 more QALYs at a cost saving of $2,034 per-patient (Table 4 in main text).

In the case where the monthly treatment completion rate is 10 percentage points lower, DOT produces additional QALYs over AiCure (0.008) and is not cost-effective with an ICER of $244,651. For 15 percentage points lower, DOT becomes cost-effective at WTP of $89,889/QALY. Further decrements to the monthly completion probability of AiCure result in DOT becoming a cost-effective alternative at all reasonable WTP thresholds.

***Secondary***

In our secondary scenario analyses we estimated non-completion related “best” and “worst” case scenarios for AiCure and DOT. For instance, the “best case” scenario would set input parameters (outside of transition probabilities) to either upper or lower bounds based on how they impacted NMB of AiCure in one-way DSA. The worst case scenario would fix these same parameters at the opposite bound in an attempt to minimize AiCure NMB. The AiCure worst case scenario also included a seven-minute dose review time for AiCure. In this exercise, we assumed transition probabilities to be fixed to their base case values. Examining these scenarios provides an extreme case analysis of AiCure performance given our data.

Under AiCure worst case and DOT best case per-patient cost-savings under DOT were $3,672 at a slight QALY decrement of 0.01, resulting in an ICER for AiCure treatment of $433,646. Under the AiCure best case and DOT worst case, AiCure was dominant and produced 0.03 additional QALYs at a cost-savings per-patient of $7,603 (see Table 4 in main text).

**Figure S1.** Results of one-way sensitivity analyses on net monetary benefits of AiCure (tornado diagram).

**
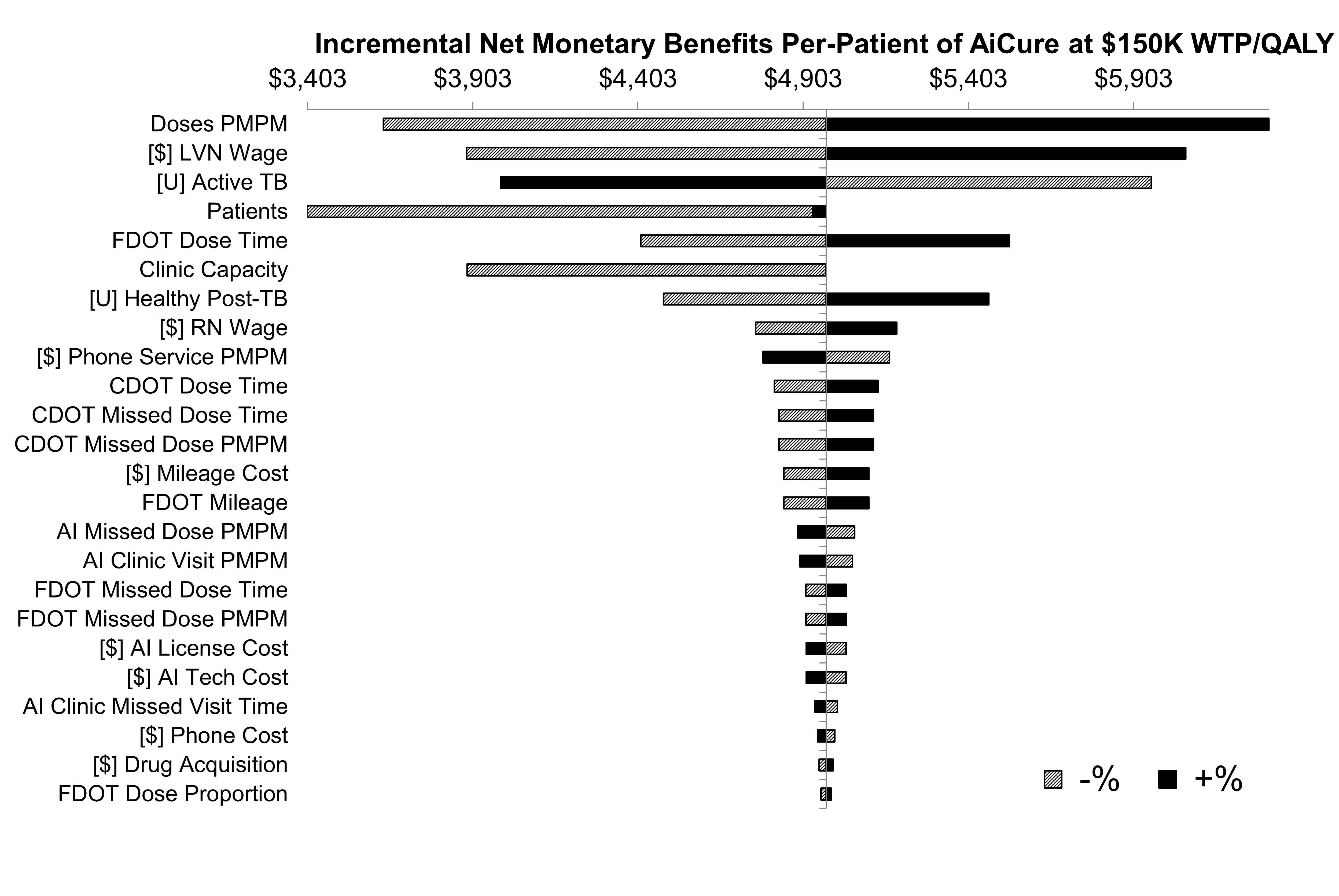
**

WTP, willingness-to-pay; QALY, quality-adjusted life year; LVN, licensed vocational nurse; TB, tuberculosis; PMPM, per-member per-month; FDOT, field directly observed therapy; AI, AiCure; CDOT clinic directly observed therapy; RN, registered nurse.

**Table S1. Transition probabilities over time by treatment arm**

| **Days on treatment** | | **Continuation** | | **Success** | | **Default^a^** | |
| --- | --- | --- | --- | --- | --- | --- | --- |
|  | | **DOT** | **AiCure** | **DOT** | **AiCure** | **DOT** | **AiCure** |
| t ≤ 120 | 1.000 | | 0.975 | 0.000 | 0.000 | 0.000 | 0.025 |
| 120 < t ≤ 150 | 0.952 | | 1.000 | 0.000 | 0.000 | 0.048 | 0.000 |
| 150 < t ≤ 180 | 1.000 | | 0.923 | 0.000 | 0.051 | 0.000 | 0.026 |
| 180 < t ≤ 210 | 0.783 | | 0.694 | 0.217 | 0.306 | 0.000 | 0.000 |
| 210 < t ≤ 240 | 0.851 | | 0.880 | 0.149 | 0.120 | 0.000 | 0.000 |
| 240 < t ≤ 270 | 0.950 | | 0.955 | 0.050 | 0.045 | 0.000 | 0.000 |
| 270 < t ≤ 300 | 0.316 | | 0.476 | 0.684 | 0.524 | 0.000 | 0.000 |
| 300 < t ≤ 330 | 0.917 | | 0.400 | 0.083 | 0.600 | 0.000 | 0.000 |
| 330 < t ≤ 360 | 0.909 | | 0.750 | 0.091 | 0.250 | 0.000 | 0.000 |
| 360 < t ≤ 390 | 0.400 | | 0.667 | 0.600 | 0.333 | 0.000 | 0.000 |
| 390 < t ≤ 420 | 0.500 | | 0.500 | 0.500 | 0.500 | 0.000 | 0.000 |
| 420 < t ≤ 450 | 0.500 | | 0.000 | 0.500 | 1.000 | 0.000 | 0.000 |
| 450 < t ≤ 480 | 0.000 | | 0.000 | 1.000 | 1.000 | 0.000 | 0.000 |

^a^ Default is defined as patients who refused continued treatment, were lost to follow-up, or went off protocol for medical reasons during our observation period

**Table S2. Distributions and parameters used in probabilistic sensitivity analysis**

| **Description** | **Arm** | **Range** | **Distribution** |
| --- | --- | --- | --- |
| **General Parameters** |  |  |  |
| Patients | Both | (10, 150) | Uniform(10, 150) |
| Clinic capacity | Both | (10, 150) | Uniform(10, 150) |
| Doses, monthly | Both | (5.5, 16.5) | Uniform(5.5, 16.5) |
| FDOT proportion | DOT | (0.68, 0.71) | Beta(3872, 1707) |
| FDOT appointment time | DOT | (0.58, 1.08) | Uniform(0.58, 1.08) |
| FDOT miles, per appointment | DOT | (3.80, 11.40) | Uniform(3.80, 11.40) |
| FDOT missed appointment time | DOT | (0.33, 0.5) | Uniform(0.33, 0.5) |
| FDOT missed appointment frequency | DOT | (2, 3) | Uniform(2, 3) |
| CDOT appointment time | DOT | (0.13, 0.38) | Uniform(0.13, 0.38) |
| CDOT missed appointment time | DOT | (0.08, 0.25) | Uniform(0.08, 0.25) |
| CDOT missed appointment frequency, monthly | DOT | (1.3, 3.9) | Uniform(1.3, 3.9) |
| AI missed dose time | AI | (0.17, 0.25) | Uniform(0.17, 0.25) |
| AI missed dose frequency, monthly | AI | (0.65, 1.95) | Uniform(0.65, 1.95) |
| AI appointment frequency, monthly | AI | (1, 2) | Uniform(1, 2) |
| **Baseline Probabilities^a^** |  |  |  |
| P(Cont., Comp., Def. \| t ≤ 120) | DOT | - | Dirichlet(63, 0, 0) |
| P(Cont., Comp., Def. \| 120 < t ≤ 150) | DOT | - | Dirichlet(60, 0, 3) |
| P(Cont., Comp., Def. \| 150 < t ≤ 180) | DOT | - | Dirichlet(60, 0, 0) |
| P(Cont., Comp., Def. \| 180 < t ≤ 210) | DOT | - | Dirichlet(47, 13, 0) |
| P(Cont., Comp., Def. \| 210 < t ≤ 240) | DOT | - | Dirichlet(40, 7, 0) |
| P(Cont., Comp., Def. \| 240 < t ≤ 270) | DOT | - | Dirichlet(38, 2, 0) |
| P(Cont., Comp., Def. \| 270 < t ≤ 300) | DOT | - | Dirichlet(12, 26, 0) |
| P(Cont., Comp., Def. \| 300 < t ≤ 330) | DOT | - | Dirichlet(11, 1, 0) |
| P(Cont., Comp., Def. \| 330 < t ≤ 360) | DOT | - | Dirichlet(10, 1, 0) |
| P(Cont., Comp., Def. \| 360 < t ≤ 390) | DOT | - | Dirichlet(4, 6, 0) |
| P(Cont., Comp., Def. \| 390 < t ≤ 420) | DOT | - | Dirichlet(2, 2, 0) |
| P(Cont., Comp., Def. \| 420 < t ≤ 450) | DOT | - | Dirichlet(1, 1, 0) |
| P(Cont., Comp., Def. \| 450 < t ≤ 480) | DOT | - | Dirichlet(0, 1, 0) |
| P(Cont., Comp., Def. \| t ≤ 120) | AI | - | Dirichlet(39, 0, 1) |
| P(Cont., Comp., Def. \| 120 < t ≤ 150) | AI | - | Dirichlet(39, 0, 0) |
| P(Cont., Comp., Def. \| 150 < t ≤ 180) | AI | - | Dirichlet(36, 2, 1) |
| P(Cont., Comp., Def. \| 180 < t ≤ 210) | AI | - | Dirichlet(25, 11, 0) |
| P(Cont., Comp., Def. \| 210 < t ≤ 240) | AI | - | Dirichlet(22, 3, 0) |
| P(Cont., Comp., Def. \| 240 < t ≤ 270) | AI | - | Dirichlet(21, 1, 0) |
| P(Cont., Comp., Def. \| 270 < t ≤ 300) | AI | - | Dirichlet(10, 11, 0) |
| P(Cont., Comp., Def. \| 300 < t ≤ 330) | AI | - | Dirichlet(4, 6, 0) |
| P(Cont., Comp., Def. \| 330 < t ≤ 360) | AI | - | Dirichlet(3, 1, 0) |
| P(Cont., Comp., Def. \| 360 < t ≤ 390) | AI | - | Dirichlet(2, 1, 0) |
| P(Cont., Comp., Def. \| 390 < t ≤ 420) | AI | - | Dirichlet(1, 1, 0) |
| P(Cont., Comp., Def. \| 420 < t ≤ 450) | AI | - | Dirichlet(0, 1, 0) |
| P(Cont., Comp., Def. \| 450 < t ≤ 480) | AI | - | Dirichlet(0, 0, 0) |
| **Utilities (QALY weights)** |  |  |  |
| Active tuberculosis | Both | (0.892, 0.992) | Uniform(0.892, 0.992) |
| Healthy, post treatment | Both | (0.563, 0.763) | Uniform(0.563, 0.763) |
| **Costs** |  |  |  |
| Licensed vocational nurse, hourly | Both | ($17.73, 53.18) | Uniform($17.73, $53.18) |
| Registered nurse, hourly | Both | ($39.63, 118.89) | Uniform($39.63, $118.89) |
| Mileage, per mile | Both | ($0.27, 0.80) | Uniform($0.27, $0.80) |
| Licensing per 50 patients, monthly | AI | ($375, 1125) | Uniform($375, $1125) |
| Phone | AI | ($26, 78) | Uniform($26, $78) |
| Phone service, monthly | AI | ($23.75, 71.25) | Uniform($23.75, $71.25) |
| Technical support, monthly | AI | ($750, 2250) | Uniform($750, $2250) |
| Pharmaceutical acquisition, monthly | Both | ($87.08, 261.24) | Uniform($87.08, $261.24) |

^a^ Ranges are omitted for parameters drawn from multivariate distributions
